# Biogenesis and function of exosome lncRNAs and their role in female pathological pregnancy

**DOI:** 10.3389/fendo.2023.1191721

**Published:** 2023-09-07

**Authors:** Min Wang, Lianwen Zheng, Shuai Ma, Ruixin Lin, Jiahui Li, Shuli Yang

**Affiliations:** ^1^ Department of Obstetrics and Gynecology, The Second Hospital of Jilin University, Changchun, China; ^2^ Department of Hepato-Biliary-Pancreatic Surgery, The Second Hospital of Jilin University, Changchun, China

**Keywords:** exosome lncRNA, pregnancy, pre-eclampsia, diabetes, gestational, abortion, biomarkers

## Abstract

Preeclampsia, gestational diabetes mellitus, and recurrent spontaneous abortion are common maternal pregnancy complications that seriously endanger women’s lives and health, and their occurrence is increasing year after year with a rejuvenation trend. In contrast to biomarkers found freely in tissues or body fluids, exosomes exist in a relatively independent environment and provide a higher level of stability. As backbone molecules, guidance molecules, and signaling molecules in the nucleus, lncRNAs can regulate gene expression. In the cytoplasm, lncRNAs can influence gene expression levels by modifying mRNA stability, acting as competitive endogenous RNAs to bind miRNAs, and so on. Exosomal lncRNAs can exist indefinitely and are important in intercellular communication and signal transduction. Changes in maternal serum exosome lncRNA expression can accurately and timely reflect the progression and regression of pregnancy-related diseases. The purpose of this paper is to provide a reference for clinical research on the pathogenesis, diagnosis, and treatment methods of pregnancy-related diseases by reviewing the role of exosome lncRNAs in female pathological pregnancy and related molecular mechanisms.

## 1 Introduction

Exosomes are membrane vesicles that are released into the extracellular fluid by various cells (1). The exosome membrane is high in cholesterol, sphingolipids, and other components, and it contains a variety of proteins, mRNA, and lncRNA, including mother cell-specific proteins and exosome-associated proteins. Exosomes are the best markers for determining the levels of various substances expressed within the mother cell (2). Scholars have paid close attention to the non-coding RNAs found in the non-coding region in recent years (3). lncRNAs are expressed in low amounts in cells or tissues and have a length of more than 200 nt. Their regulation primarily consists of epigenetic regulation, transcriptional regulation, and post-transcriptional regulation, all of which affect cell proliferation, apoptosis, and differentiation and play an essential role in the development of many diseases (4, 5). Research has found that lncRNA plays an important role in the homeostasis of cells or tissues during development. Although LncRNAs cannot directly regulate protein translation, they can exert regulatory power through miRNAs, which may be mediated as a mediator (6). Some lncRNAs are enriched in exosomes, while others are almost absent, implying that lncRNAs are selectively sorted into exosomes (7). Pathological pregnancy is becoming more common, and the resulting problem of reduced fertility cannot be ignored (8). Exosomal lncRNA, which is abundant and stable in plasma and has high ribonuclease activity, can serve as a reference for clinicians in the diagnosis of pregnancy-related disorders (9). As a result, it is clinically significant to investigate the exosome lncRNAs that affect women’s pathological pregnancy behavior, as this can help to further investigate the disease’s development mechanism and provide new ideas and strategies for disease treatment, thereby genuinely protecting women’s reproductive health (10).

## 2 Overview of exosomes

### 2.1 Biogenesis of exosomes

**Figure 1:**
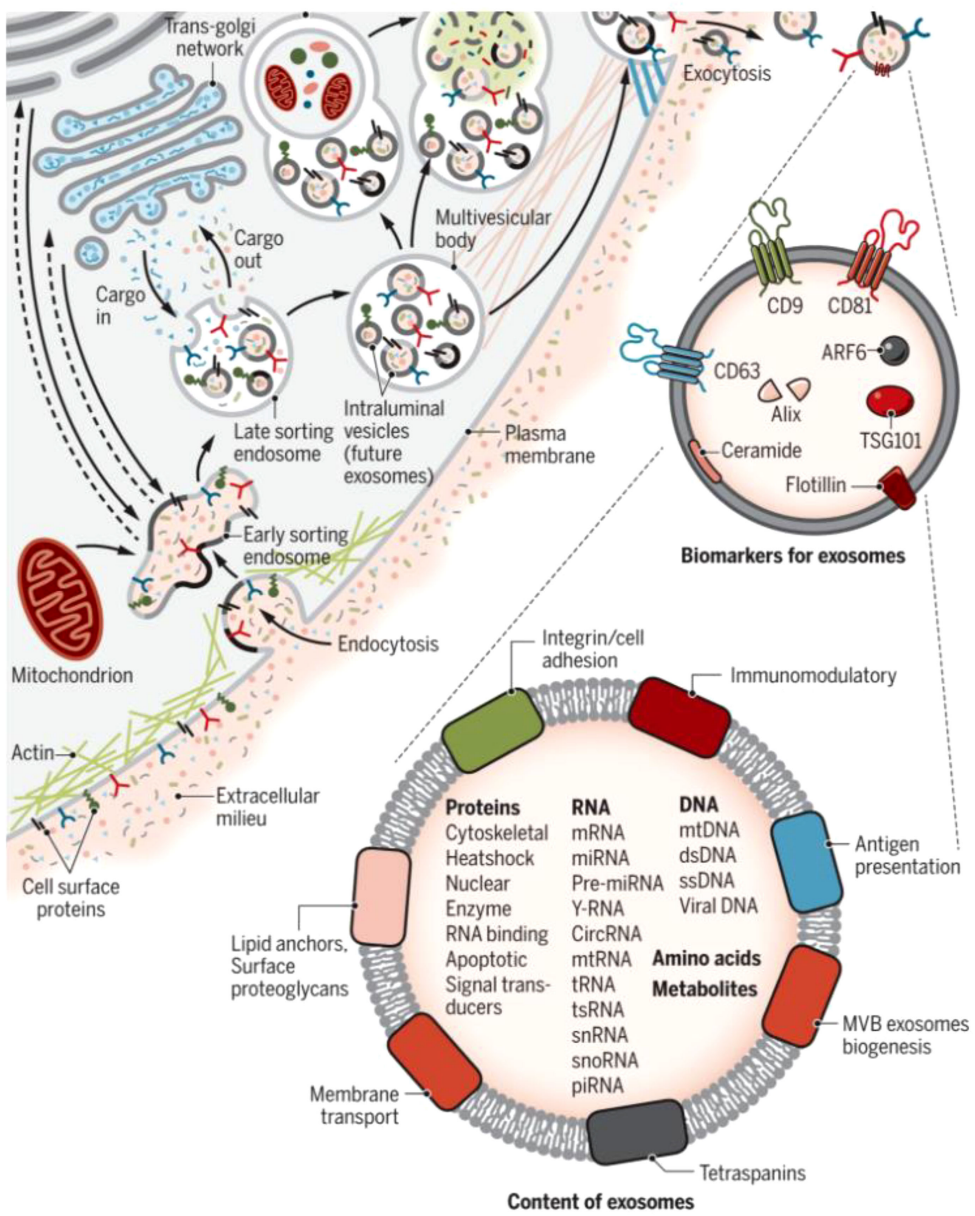
Biogenesis and identification of exosomes. Fluid and extracellular constituents such as proteins, lipids, metabolites, small molecules, and ions can enter cells, along with cell surface proteins, through endocytosis and plasma membrane invagination. The resulting plasma membrane bud formation in the luminal side of the cell presents with outside-in plasma membrane orientation. Several proteins are implicated in exosome biogenesis and include ESCRT proteins, as well as others that are also used as markers for exosomes (CD9, CD81, CD63, flotillin, ceramide, and Alix). Exosome surface proteins include tetraspanins, integrins, immunomodulatory proteins, and more. Exosomes can contain different types of cell surface proteins, intracellular proteins, RNA, DNA, amino acids, and metabolites (15).

Exosomes are made up of a double-layered lipid membrane structure that ranges in size from 30nm to 150nm and contains DNA, mRNA, and lncRNA (11, 12). Exosomes are present in almost all eukaryotic body fluids (13), including uterine fluid, urine, amniotic fluid, breast milk, peritoneal fluid, and cell culture fluid, according to recent research (14). When exposed to extracellular stimuli, microbial attack, or other stress conditions, the cell membrane invaginates, allowing material from outside the cell membrane to enter the cell along with the cell membrane surface proteins, resulting in the formation of the early-sorting endosome (ESE) (15). By “budding,” the ESE membrane generates multivesicular bodies (MVBs) or late endosomes. Finally, MVBs are secreted extracellularly to form exosomes under the control of the endosomal sorting complex required for transport (ESCRT) and specific proteins (16). Exosomes and target cells interact in three ways: exosome surface membrane proteins directly bind to target cell receptors, activating intracellular signaling pathways; target cells take up exosomes via endocytosis; and exosomes can directly fuse with the plasma membrane of target cells, releasing the miRNAs and lncRNAs they carry into the target cells (17) (**Figure 1**).

### 2.2 Function of exosomes

Exosomes are critical intercellular messengers that regulate cellular physiological functions such as immune regulation, cell proliferation, antigen expression and presentation, and bioenergetic conversion (18, 19). Exosomes transport nucleic acids, which play an essential role in cellular communication (20). Exosomes contain at least ten different types of RNA, and actively secreted exosomes can package a large amount of intracellular information material for transmission from one cell to another, achieving cross-cellular regulation and participating in intercellular communication and microenvironment regulation (21). Because cell membrane transmembrane proteins are also expressed on the exosome membrane, it is critical for exosome identification (22). Glycoproteins and transmembrane proteins are enriched in intercellular communication and adhesion events, which can be utilized to determine their cellular or tissue origin, such as placental-derived exosomes that express placental-like alkaline phosphatase (PLAP) (23). Exosomes can control morphogenetic signaling, immune cell recruitment, and genetic material transport to carry out a range of cell biological tasks in the cellular microenvironment (24). The majority of methods for detecting exosomal nucleic acid information rely on the presence of mRNA and microRNA in exosomes (25). In recent years, the importance of targeting exosomal lncRNAs has gained more attention. LncRNAs protected by the exosomal tegument exhibit higher expression and better stability than lncRNAs isolated directly from peripheral bodily fluids, and their results are trustworthy.

## 3 Overview of lncRNAs

### 3.1 lncRNAs play a role in the regulation of pre-transcription

**Figure 2:**
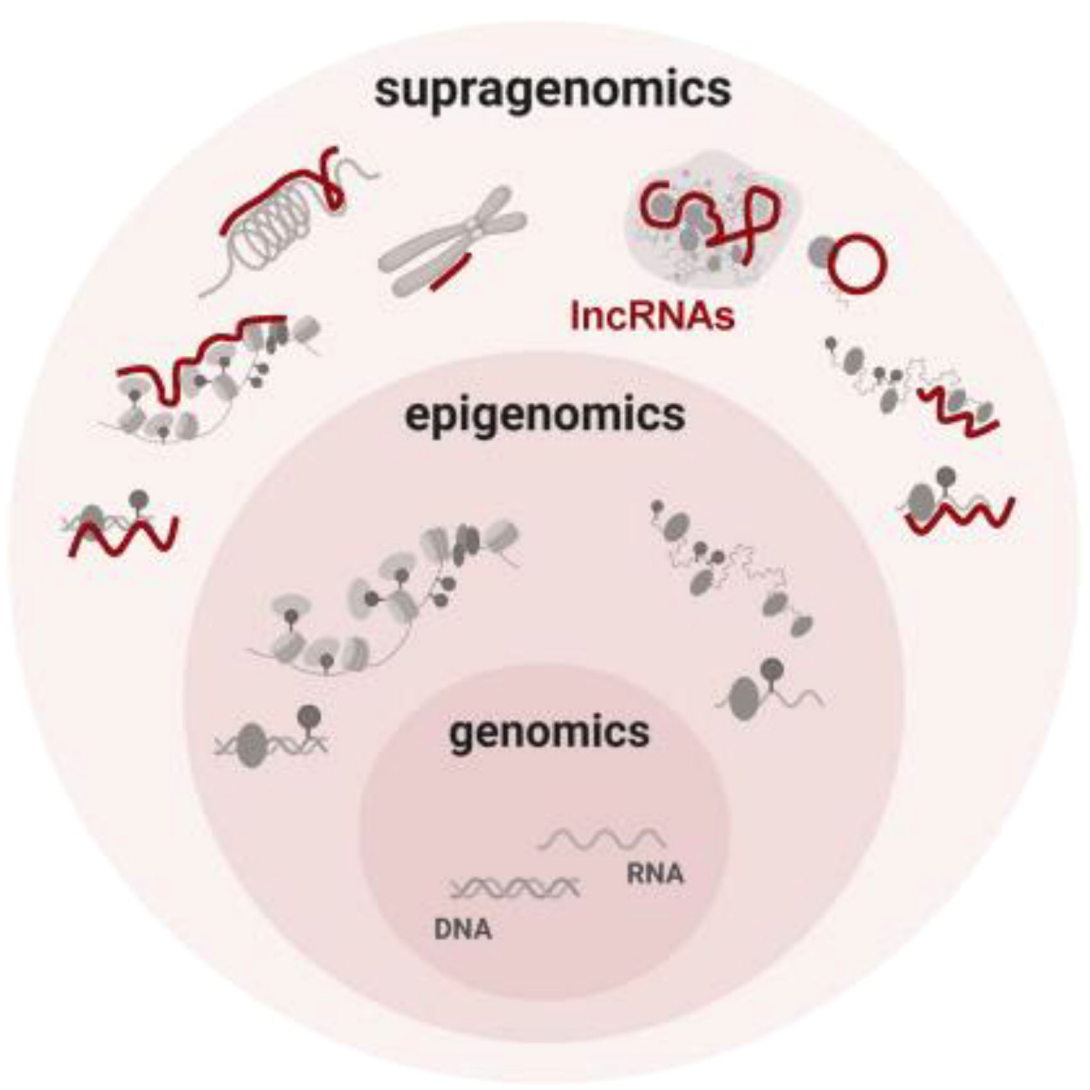
Conceptual summary of supragenomic regulation by lncRNAs Vast and heterogeneous lncRNAs interact broadly with gene regulatory machineries. By providing a supragenomic layer of control built upon genomic and epigenomic processes, lncRNAs modulate many levels of gene regulation, from transcription to protein modification (26).

By controlling the regulation of target genes by the epimodification complex before transcription takes place through chromatin modification, genomic imprinting, and dosage compensation effects, lncRNAs in the nucleus play an epigenetic function in gene expression (26, 27). The chromatin state and the way proteins attach to chromatin are both altered by the different ways that lncRNAs can modify histones, including methylation, acetylation, and ubiquitination. In order to interact with the histone modification complex Polycomb Repressive Complex 2 (PRC2) and mediate histone methylation and demethylation, LncRNA HOX antisense intergenic RNA (HOTAIR) may serve as a molecular scaffold. The ATP-dependent chromatin remodeling complex plays a major role in controlling chromatin remodeling, an enzymatic co-process that enables nucleosomal DNA acquisition by altering the structure, composition, and placement of nucleosomes. BRG1, the central component of the chromatin remodeling complex SWI/SNF, can interact with lncRNA MANTIS, stabilizing it. An open chromatin conformation is induced by the interaction of BRG1 with BAFl55, another chromatin remodeling factor, and this increases the transcription of genes involved in angiogenesis. MANTIS is a lncRNA that is thought to have pro-endothelial angiogenic potential (**Figure 2**).

### 3.2 LncRNAs play a role in controlling gene transcription

By preventing the RNA Pol II complex from attaching to the promoter, LncRNA can interfere with transcription (28). According to Latos et al., the lncRNA Aim transcript covers the lgf2r promoter region and prevents the recruitment of RNA Pol II, preventing the transcription of lgf2r. LncRNA can directly activate the downstream genes by bridging the gap between the enhancer and promoter regions (29). It is crucial for the transcriptional activation of Snail that activating lncRNAs interact with enhancers and transcription factors, be present inside the enhancer, and have enhancer activity. Studies demonstrate that the binding of activating lncRNAs to the Snail promoter region is mediated by the transcriptional co-activation complex Mediator, acting in concert with CDK8-catalyzed histone modifications to promote transcriptional activation of Snail, underscoring the significance of activating lncRNAs in human disease. lncRNA LNMAT2 is loaded into exosomes through direct interaction with heterogeneous ribonucleoprotein A2B1 (hnRNPA2B1) by direct interaction to exosomes and delivery to human lymphatic vessel endothelial cells (HLEC); subsequently, LncRNALNMAT2 forms a triple complex with the PROX1 promoter by inducing hnRNPA2B1-mediated H3K4me3 and enhances PROX1 transcription, thereby promoting lymphangiogenesis and lymph node metastasis.

### 3.3 LncRNAs participate in the selective shear regulation process

Genetic information from DNA is translated into mature mRNA by biological processes, including shearing and splicing; this process does not function directly (30). More than 95% of gene transcripts go through a process called selective splicing, which makes biological gene expression more complex and plays a regulatory function in the growth and development of living things (31). LncRNA participates in gene expression regulation by constructing different variable splicing forms through splicing factors, regulating miRNA precursor transcripts and upstream differentially methylated regions (DMR) (32). In order to control the phosphorylation level of the serine/arginine-rich (SR) protein family, which controls mRNA splicing, lncRNAs have been shown to function as regulatory factors (33). It has been demonstrated that the lncRNA metastasis-associated lung adenocarcinoma transcript 1 (MALAT1) associates with numerous proteins to form a nuclear speckle that takes part in varied pre-mRNA shearing (34). Vidisha et al. discovered that the lncRNA MALAT1 selectively binds to the nuclear protein TDP-43, resulting in the recruitment of the SR protein family to the nucleus. The nuclear speckle was recruited by splicing factors like TDP-43, which improved the SR protein family’s capacity to splice and thus raised its level of phosphorylation.

## 4 Exosomal lncRNA offers excellent clinical use potential

Specificity, as exosomes include particular indicators of tissue or cellular origin, is one of the properties of exosome-derived lncRNAs as biomarkers. Notably, the RNase activity is high in the extracellular environment, but extracellular ncRNAs remain relatively stable in plasma, suggesting that circulating ncRNAs may be protected and circumvented from harsh conditions (35). Exosome stability: Exosomes remain in a stable state in bodily fluids, and RNA is not significantly exposed because of the protection of lipid bilayer membranes, where enzymes cannot easily digest the contents of exosomes (36). Although the lncRNA expression is low in some cells, it is highly expressed in their secreted exosomes and correlates with the development of disease. Exosomes are widely distributed in different body fluids. The primary techniques for isolating exosomes are gradient density centrifugation, differential ultracentrifugation, polymer immunoprecipitation, gel exclusion separation, and membrane affinity kits (37, 38). Exosomal lncRNAs combine the benefits of exosomes and lncRNAs in a way that enhances the effectiveness of treatment and the prognosis of patients (39). Exosomes can be employed as specialized targets for treating disease. As a result, the non-invasive detection of lncRNA produced from exosomes has the potential to be exploited as a biomarker for future diagnosis and therapy (40).

## 5 Correlation of exosomal lncRNA with pregnancy

### 5.1 Endometrial tolerance

**Figure 3:**
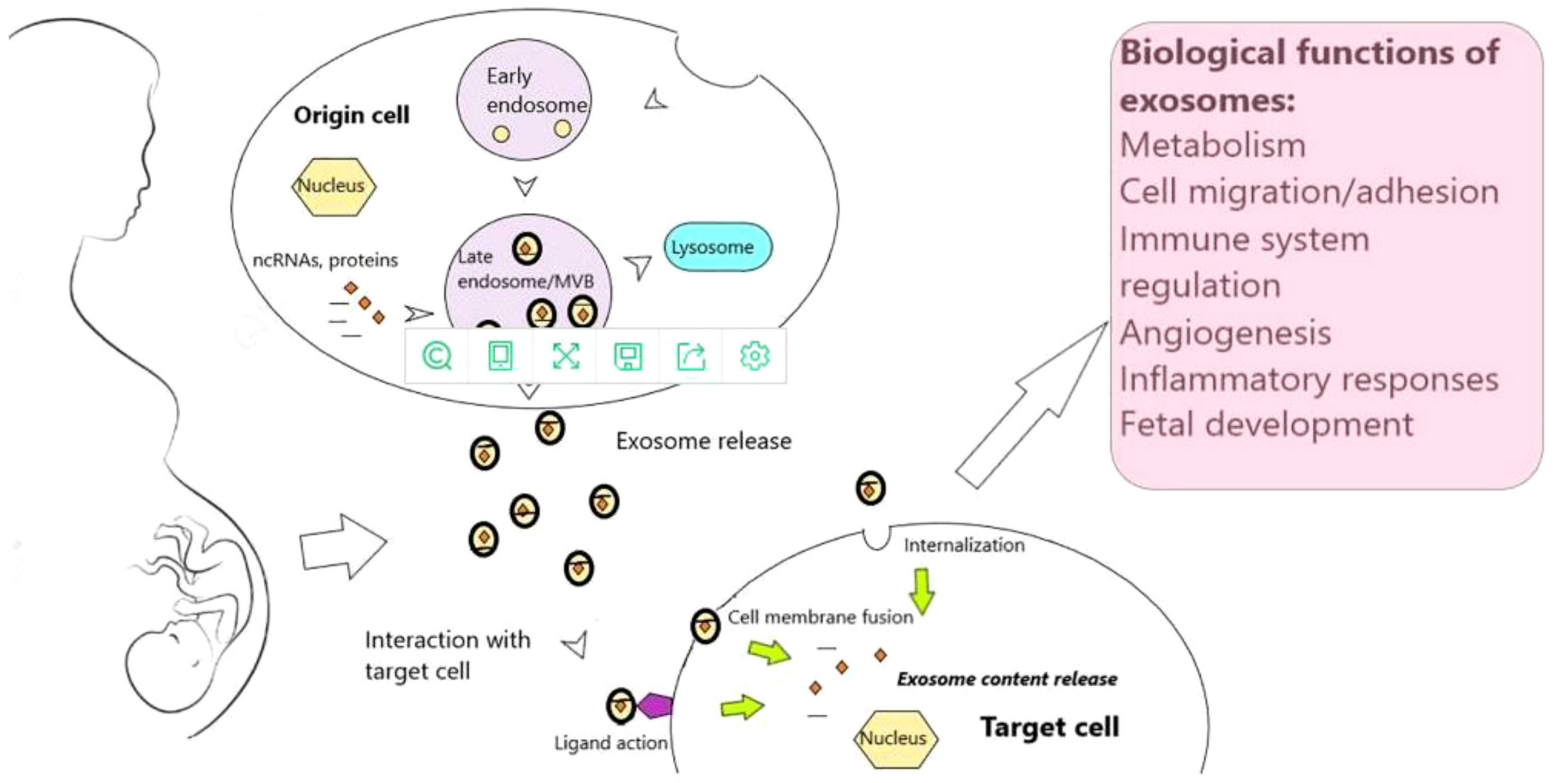
Exosomes and exosomal noncoding RNAs throughout human gestation (9).

Fertilization, implantation, endometrial metaphase, placental development, and birth are significant, complex, and irreversible aspects of pregnancy in humans and other mammals (41). Abnormalities or the failure of any one of these processes can have an impact on the pregnancy’s outcome (9) (**Figure 3**). By directly influencing embryonic development and regulating the expression of important adhesion molecules, the endometrium can leak exosomes into the uterine fluid and govern implantation (42). h19 is a naturally occurring lncRNA that is widely produced, developmentally controlled, and affects Let-7 target genes (43). Reduced expression of the H19 gene and the ITG-3 protein was found in the recurrent implantation failure (RIF) group, proving that the expression of the lncRNAH19 is positively associated with that of the ITG-3 protein, reducing endometrial tolerance and ultimately causing implantation failure.lncRNATUNAR was initially expressed in the human endometrium and is thought to play a role in embryo implantation by controlling the attachment of blastocysts to the endometrial epithelium as well as the proliferation and ecdysis of embryonic stem cells. In healthy females, the expression of lncRNATUNAR was increased in LH+2 and downregulated in LH+7. Due to the cyclic expression of the endometrium and its abnormal expression in RIF patients, lncRNATUNAR may have a role in controlling the embryonic implantation process. lncRNATUNAR was found to be increased in LH+7 endometrium from RIF patients.

### 5.2 Promote the establishment of immune tolerance at the maternal-fetal interface

The maternal-fetal interface, which is made up of extra-embryonic tissues and the meconium, is a crucial component that helps the mother’s immune system adapt to the fetus during pregnancy (44). By triggering the JNK and p38 signaling pathways in meconium macrophages via the exosomal lncRNA Zinc finger E-box-binding homeobox 2 antisense RNA 1 (ZEB2-AS1), trophoblast cells can create and maintain the maternal-fetal immune tolerance microenvironment. This promotes the polarization of macrophages toward the inhibitory M2 phenotype. In order to support the orderly development of a typical pregnancy, the induced M2 can also operate on trophoblast cells and encourage their proliferation and differentiation at the same time. The decline in recurrent spontaneous abortion patients’ metaphase macrophage M2 may be brought on by low expression of ZEB2-AS 1 in their trophoblast exosomes. The preservation of a stable pregnancy may be made possible by apoptosis, which may allow the human placenta and fetal allografts to avoid a detrimental maternal immune attack while pregnant and enjoy immunological privileges in the uterine cavity (45). The results point to functional FasL and TRAIL being secreted by human early and term placentas, which help deliver apoptosis and shield the fetal placenta from activated maternal immune cells. This suggests placental exosome-mediated fetal immunological privilege. Exosomes play a key role in preserving homeostasis at the maternal-fetal interface throughout pregnancy and are a cutting-edge instrument for intercellular communication (46).

### 5.3 Promote successful embryo implantation

Embryo implantation, a crucial stage in pregnancy, is the process by which the blastocyst interacts with the uterus in a receptive condition while in an activated state before making close contact with the endometrium (47). Mammalian reproduction depends on the embryo’s successful implantation into the mother (48). Placenta-derived exosome (PEXO) can be ingested by epithelial and stromal cells in the meconium, changing the regional immunological milieu, according to *in vitro* research. In order to start and maintain pregnancy, cell-to-cell communication at the maternal-fetal interface is crucial. Exosomes, which the embryo secretes to help with implantation, enhance the embryo’s natural adaptability and support successful implantation and the start of pregnancy, allowing the embryo to control its growth. Before embryo implantation, extracellular vesicles of decidual epithelial cells can activate the expression of Bcl2, Bax, Casp3, and Tp53 genes in endometrial epithelial cell apoptosis pathways. Exosomes primarily increase the expression of endometrial epithelial cell adhesion-related proteins following embryo implantation to aid subsequent adherence (49). Exosomes from human ectodermal stromal cells can upregulate the production of trophoblast calmodulin and so boost invasive activity in addition to that of epithelial cells. They can also promote the creation of endothelial cell tubes and may be crucial for angiogenesis. In mouse trials, embryos treated with embryonic-derived exosomes were able to increase implantation rates and improve implantation ability. They could also improve blastocyst formation rate, embryo quality, and future growth and development.

## 6 Exosomal lncRNA and pathological pregnancy

Exosomes contain various proteins and nucleic acids, serving as diagnostic markers for obstetric diseases with high specificity. The study of exosome lncRNA can explore the pathogenesis of various diseases in pathological pregnancy, screen biomarkers, and provide a new basic basis for the diagnosis and treatment of diseases. Many lncRNAs associated with tumor cell function may also play significant regulatory roles for trophoblasts because placental trophoblasts share characteristics with tumor cells during proliferation, migration, and invasion (50). This is especially true for pathways involved in angiogenesis, cell cycle regulation, cell migration, and invasion (51). Through interacting with miR-216a-5p and controlling recombinant hexokinase 2(HK2), LncRNA MALAT1 prevents placental trophoblast growth, migration, and invasion, as well as angiogenesis, cell cycle arrest, and apoptosis. In addition to the syncytial trophoblast-specific protein PLAP and the trophoblast-specific protein human leukocyte antigen G(HLAG), PEXO is abundant in the exosomal marker proteins CD9, CD63, and CD81 (52–54). These two proteins can be separated to form PEXO in maternal peripheral blood, and the quantity of PEXO can be used to forecast fetal growth and ascertain the success of a pregnancy. Exosomes carry a variety of payloads in maternal peripheral blood, and histological study of these exosomes in various disorders has shown that changes in their type and amount may negatively impact the function of target cells (55).

### 6.1 Exosomal lncRNA and Preeclampsia

#### 6.1.1 Exosomes participate in the occurrence of PE

A key contributor to increased maternal and neonatal mortality, Preeclampsia is a pregnancy problem that manifests beyond 20 weeks of gestation with proteinuria, hypertension, or other systemic damage (56–60). To lower maternal and neonatal mortality and enhance mother and baby health, early Preeclampsia diagnosis is crucial (61). The number of placental exosomes, changes in their composition, and their impact on the maternal immune system are thought to be the key ways that PEXO contributes to Preeclampsia pathogenesis (62).

Preeclampsia patients exhibit decreased expression of functional proteins such as matrix metalloproteinase (MMP) 2 and MMP9, as well as increased levels of phosphatidylserine (PS) and lower levels of phosphatidic acid and phosphatidylglycerol in exosomes when compared to the normal pregnancy group (63, 64). The remodeling of spiral arteries, fetal growth, superficial placentation, reduced blood flow, and ultimately the development of Preeclampsia can all be impacted by decreased expression of any one of these proteins. Human umbilical cord mesenchymal stem cells (HUCMSCs)- exosomes can increase IL-10, TNF-, IFN-, and the local recruitment of NK cells and macrophages *in utero*, modulating the immunological balance at the maternal-fetal plane and indirectly affecting pregnancy outcomes. Compared to women who had normal pregnancies, the placentas of Preeclampsia patients had a significant number of differently expressed lncRNAs, according to research using lncRNA microarray technology. They were implicated in the development of Preeclampsia by interfering with trophoblast cell activity, among other things (65).

#### 6.1.2 Decreased expression of exosomal lncRNA in Preeclampsia

A lncRNA called MALAT1 is linked to placental implantation and penetration (66). When compared to healthy pregnant women, Preeclampsia sufferers’ placental tissues express less of the lncRNA MALAT1 (67). When lncRNAMALAT1 levels are low, EMT is induced with less trophoblast invasion, migration, and angiogenesis, which can result in a higher uterine spiral artery remodeling injury (68). According to research, lncRNA MALAT1 levels in plasma exosomes from pregnant women with Preeclampsia were positively correlated with vascular endothelial growth factor (VEGF) expression levels. This suggests that downregulating lncRNA MALAT1 levels in plasma exosomes may speed up the progression of Preeclampsia by controlling VEGF expression, which in turn suppresses angiogenesis (69). Wu et al. discovered that lncRNAMALAT1 could bind to miR-206, prevent the latter from degrading IGF-1 mRNA, boost IGF-1 expression, and activate the PI3K/AKT signaling pathway, which in turn encouraged trophoblast migration and invasion (70).

Preeclampsia patients had a lower placental expression of the short nucleolar RNA host gene 22 (SNHG22) than healthy pregnant women did. By interacting with miR-128-3p to encourage PCDH11X expression and open up downstream pathways, lncRNASNHG22 can have a role in Preeclampsia. In comparison to normal pregnant placental tissues, the expression of lncRNA XIST was found to be considerably reduced in the placentas of Preeclampsia patients. lncRNA XIST is involved in the development of Preeclampsia by regulating the proliferation and invasive ability of trophoblast HTR-8/SVneo through miR-135b. The lncRNA TUG1 was downregulated in the placental tissue of Preeclampsia patients compared to healthy pregnant women, and this downregulation decreased cell proliferation, migration, and invasion while promoting trophoblast death. While TUG1 downregulation boosted the expression of the enhancer of zeste homolog 2 (EZH2) and decreased the levels of the Rho family GTPase 3 (RND3) in Preeclampsia, it prevented remodeling of the uterine spiral artery. Studies have shown that downregulating lnc-dendritic cell (DC), a lncRNA expressed in DC, prevents monocytes from differentiating into DC, diminishing the inhibitory effect of DC on Treg, encouraging the proliferation of Th1 cells in the meconium of Preeclampsia patients, and ultimately promoting Preeclampsia (71, 72).

#### 6.1.3 Increased expression of exosomal lncRNA in Preeclampsia

Compared to normal pregnancies, the placenta of Preeclampsi patients has higher levels of lncRNAH19 expression (73). In the human choriocarcinoma cell line JEG-3 and the human choriocapillaris trophoblast cell line HTR-8/SVneo, lncRNAH19 regulates the PI3K/Akt/mTOR pathway and boosts autophagy and invasiveness (74). Moreover, the lncRNAH19 gene encodes miR-675, which can suppress cell growth by lowering the expression of the nodal modulator 1 (NOMO1) in JEG-3 cells (75). In the placental tissues of Preeclampsia patients, lncRNA GAS5 expression is elevated, and its level rises as the severity of the disease does as well (76). The development of atherosclerosis can be aided by the lncRNA GAS5, which can encourage the death of vascular endothelial cells as well as aberrant proliferation and migration of vascular smooth muscle cells. The degree to which lncRNA GAS5 was expressed in Preeclampsia patients was inversely correlated with spiral artery lumen area and positively correlated with spiral artery wall thickness, suggesting that lncRNA GAS5 may be connected to the process of placental spiral artery recasting. Many lncRNAs have an impact on trophoblast cells’ physiological processes, which are intimately associated with the development of Preeclampsia and include migration, invasion, proliferation, and apoptosis (77). Determining the regulatory roles that Preeclampsia-related lncRNAs play in various pathways can therefore assist in clarifying the interactions that contribute to Preeclampsia pathophysiology, identify essential molecules for diagnosis and therapy, and provide potential targets for Preeclampsia prevention and treatment (78).

### 6.2 Exosomal lncRNA and gestational diabetes mellitus

#### 6.2.1 Exosomal lncRNA’s role in the emergence of gestational diabetes mellitus

The condition known as gestational diabetes mellitus is characterized by aberrant glucose metabolism in the body, which is brought on by insulin insufficiency and hormonal changes during pregnancy (79). In extreme cases, gestational diabetes mellitus can result in maternal and neonatal death. The incidence of gestational diabetes mellitus is increasing as people’s lifestyles and diets change (80). Research has revealed that gestational diabetes mellitus is a risk factor for cardiovascular disease and type 2 diabetes (T2DM), which can raise the risk of immediate or long-term issues in expecting mothers and children and gravely jeopardize the physical and mental health of women and neonates (81, 82). Insulin resistance (IR), one of the primary causes of gestational diabetes mellitus, has a complex etiology and unknown pathophysiology (83, 84). Hence, a contemporary topic that requires attention is the quest for biomarkers with high sensitivity and specificity for the early diagnosis and treatment of gestational diabetes mellitus as well as the maternal postpartum state (85).

Exosome levels and biological activity were shown to vary with gestational stage in pregnant women with gestational diabetes mellitus and normal glucose tolerance (NGT) (86). When matched for gestational weeks, the concentration of placental exosomes in the plasma of gestational diabetes mellitus patients is significantly higher than that of healthy pregnant women and may positively correlate with baby weight. Between 22 and 28 weeks of gestation, the plasma exosomes mostly displayed altered expression of proteins related to insulin sensitivity, including CAMK2b and pregnancy-as-sociated plasma protein A (PAPPA). As a result, gestational diabetes mellitus patients’ plasma exosomes play a significant role in controlling glucose homeostasis during pregnancy (87).

#### 6.2.2 Decreased expression of exosomal lncRNA in gestational diabetes mellitus

An endogenous lncRNA called SNHG17 can bind and inhibit the transcription of miRNAs, which control the transcription and expression of target genes and contribute to the onset and progression of gestational diabetes mellitus (88). Research suggests that lncRNASNHG17, which is connected to vascular endothelial cell survival and angiogenesis, is abnormally underexpressed in the peripheral blood of T2DM patients (89). Serum lncRNASNHG17 levels are significantly lower in pregnant women with gestational diabetes mellitus than in healthy pregnant women, and they are correlated with fasting blood glucose (FBG), glycosylated hemoglobin, type A1c (HbA1c), and Homeostasis model assessment(HOMA)-IR negatively and HOMA-β positively. This suggests that lncRNASNHG17 may be involved in the pathological lesion process of gestational diabetes mellitus by influencing these variables (90). In gestational diabetes mellitus patients, the expression level of lncRNAMALAT1 was discovered to be correlated with the disease severity and to have a strong negative relationship with the maternal BMI and FBG at delivery (91). Compared to healthy pregnant women, gestational diabetes mellitus patients had significantly lower serum levels of the lncRNAMALAT1 gene (92). By increasing miR-155-5p expression, suppressing IGF2 expression, enhancing trophoblast cell survival, migration, and invasion, and reviving the biological activity of high glucose-induced trophoblast cells, *in vitro* cellular assays demonstrated that silencing lncRNAMALAT1 plays a role in the development of gestational diabetes mellitus (93).

#### 6.2.3 Increased expression of exosomal lncRNA in gestational diabetes mellitus

Maternally expressed gene 3 (MEG3) has been linked to abnormal placental expression, trophoblast migration, and apoptosis. It also has an impact on the expression of the NF-B, caspase-3, and Bax proteins in the placenta. Human umbilical vein endothelial cells (HUVEC) from gestational diabetes mellitus have elevated MEG3 expression, which affects fetal endothelial function through the PI3K signaling pathway (94). MEG3 overexpression, meanwhile, was able to prevent human villous trophoblast HTR-8/SVneo from proliferating, migrating, and invading while inducing apoptosis, indicating that MEG3 may be implicated in the development of gestational diabetes mellitus and playing a significant role (95). The conserved family SNX member sortingnexin17 (SNX17) is crucial for the endocytic, intracellular transport of cell surface proteins. It is crucial for endocytosis and the intracellular activities that involve cell surface proteins. It was discovered that lncRNA-SNX17 was elevated and miR-517a was downregulated in the blood of gestational diabetes mellitus patients and that the two together were more useful for the diagnosis of gestational diabetes mellitus than the single index test (96). Both the lncRNA P21 and the lncRNA H19 were shown to be elevated in the serum and placental tissues of gestational diabetes mellitus patients. These two lncRNAs may cooperate to promote the development of gestational diabetes mellitus and correlate with newborns’ birth weights. The incidence of gigantic newborns in gestational diabetes mellitus patients was connected with serum lncRNA HOXA transcript expression at the distal tip (HOTTIP), which was considerably increased in gestational diabetes mellitus patients. Both miR-21 and lncRNA HOTTIP were discovered to be abnormally expressed in gestational diabetes mellitus and connected with a poor pregnancy outcome, which could be used as a prediction for early identification of gestational diabetes mellitus (97). In order to identify other potential targets for the therapy of gestational diabetes mellitus, we will keep screening exosomal lncRNAs strongly associated with IR and glucose metabolism and investigate their potential participation in regulatory networks (98, 99).

### 6.3 Exosomal lncRNA and recurrent spontaneous abortion

#### 6.3.1 The emergence of recurrent spontaneous abortion is intimately related to exosomal lncRNA

Two or more consecutive spontaneous abortions constitute the incidence of recurrent spontaneous abortion (100). Early superficial placental implantation, poor trophoblast migration and invasion, and defective placental microvascular formation are three significant pathophysiological causes for the development of recurrent spontaneous abortion, all of which are becoming more common (101, 102). Exosomal lncRNAs participate in the regulation of trophoblast invasive capacity, the expression of cyclin-dependent kinases (CDKs), and various physiological processes like lipid metabolism and protein synthesis. These actions have an impact on early embryonic implantation. Exosomes produced by mesenchymal stem cells can operate on trophoblast cells to cause them to secrete MMP, which in turn makes trophoblasts more invasive. By being endocytosed by trophoblast cells, exosomes from metaphase macrophages can carry out the corresponding biological action (103). When we co-cultured exosomes from patients with unexplained recurrent spontaneous abortion (URSA) and patients with normal early pregnancy abortion with trophoblast HTR-8/SVneo cells, we discovered that the number of cells migrating in URSA patients was significantly lower than that in patients with normal early pregnancy abortion. Both the number of cells migrating and the viability of the cells were much lower in URSA patients. This shows that meconium macrophages can control trophoblast cells’ biological behavior by secreting exosomes, leading to embryonic arrest and playing a role in the emergence of URSA (104).

The regulation of embryonic development, endometrial tolerance, trophoblast function, stimulation of inflammation, placental vascular development, and the regulation of embryonic stem cells are the key ways that lncRNAs contribute to miscarriage (105, 106). It was discovered that the p53-regulated lncRNA lncPRESS1 safeguards embryonic stem cells by inhibiting the function of the silent information regulator (SIRT) 6 (107). Meanwhile, lncKdm2b stimulates the production of transcription factor zinc finger and BTB structural domain protein 3, promoting early embryonic development and embryonic stem cell self-renewal (108). Small interfering RNA-silenced mouse embryonic stem cells may suffer harm or even miscarry if appropriate lncRNAs are administered (109). LncRNA screening before embryo implantation can lower the chance of a failed transfer and miscarriage since lncRNAs play a significant role in controlling embryonic stem cell development (110, 111).

#### 6.3.2 Exosomal lncRNA offers fresh approaches to identifying and treating recurrent spontaneous abortion

Defective gene expression and aberrant cell proliferation are brought on by the increased expression of LncRNA H19 in recurrent spontaneous abortion patients’ embryonic tissues (112). Through its binding to let-7, lncRNA H19 inhibits ITG3 expression. This has an impact on how cells adhere to the basement membrane and lowers endometrial tolerance. As a result, embryos are lost (113). Apoptosis and iron death are promoted by IncRNA H19 by downregulating the expression of Bax and upregulating the expression of Bcl2 and GPX4 in recurrent spontaneous abortion. Nuclear enriched transcript 1(NEAT1) and MALAT levels in recurrent spontaneous abortion patients are much lower than in healthy women, and trophoblast cell proliferation, migration, invasiveness, and apoptosis were all reduced when the MALAT1 gene was knocked down (114). The human plasmacytoma variant translocation 1 (PVT1) promoter is directly impacted by lncRNA regulation, which also lowers the ability of trophoblast cells to invade (115).

Patients with recurrent spontaneous abortion had increased villous tissue LINC01088 expression. ARG1 can be bound by LINC01088, which is mostly found in the nucleus of trophoblast cells (116). This increases ARG1’s protein stability and suppresses the expression of NOS. When LINC01088 is overexpressed, ARG1’s protein stability is improved, which in turn lowers the expression of NOS and lowers NO expression. The JNK/P38 MAPK signaling pathway is further activated by the decreased NO, which impairs trophoblast cell proliferation, invasion, and migration and contributes to the development of recurrent spontaneous abortion. The lnc-SLC4A1-1 gene was discovered to be significantly expressed in the villi of URSA patients and to be able to trigger an immunological response via the NF-B/CXCL8 axis (117). In peripheral blood mononuclear cells from pregnant women with URSA, the expression levels of the lncRNAs SNHG5 and KLF4 were aberrant, and both of these were risk factors for the development of URSA (118, 119). We discovered that the lncRNA types HOTAIR and SNHG7 are related to recurrent spontaneous abortion pathogenesis and control trophoblast proliferation, apoptosis, invasion, and chorionic villus angiogenesis (120, 121). These investigations revealed prospective biomarkers and therapeutic targets, offering fresh perspectives on the early detection and management of recurrent spontaneous abortion (122) (**Table 1**).

**Table 1:** The expression of exosomal lncRNAs in pathological pregnancy.

Type of disease	Exosomal lncRNAs	Expression increases/decreases	References
Preeclampsia	SNHG22	decreases	(77)
Preeclampsia	MALAT1	decreases	(67)
Preeclampsia	HIF1A-AS1	decreases	(77)
Preeclampsia	SNHG12	decreases	(77)
Preeclampsia	MVIH	decreases	(77)
Preeclampsia	GHET1	decreases	(77)
Preeclampsia	DANCR	decreases	(77)
Preeclampsia	SNHG5	decreases	(77)
Preeclampsia	TUG1	decreases	(77)
Preeclampsia	lnc-DC	decreases	(72)
Preeclampsia	H19	increases	(73)
Preeclampsia	GAS5	increases	(76)
Preeclampsia	HIF1A	increases	(77)
Preeclampsia	SH3PXD2A-AS1	increases	(77)
Preeclampsia	LINC01410	increases	(77)
Preeclampsia	INHBA-AS1	increases	(77)
Preeclampsia	RPAIN	increases	(77)
Preeclampsia	TINCR	increases	(77)
Gestational diabetes mellitus	MALAT1	decreases	(92)
Gestational diabetes mellitus	PVT1	decreases	(98)
Gestational diabetes mellitus	SNHG17	decreases	(90)
Gestational diabetes mellitus	MEG3	increases	(94)
Gestational diabetes mellitus	SNX17	increases	(96)
Gestational diabetes mellitus	P21	increases	(98)
Gestational diabetes mellitus	H19	increases	(98)
Gestational diabetes mellitus	HOTTIP	increases	(97)
Gestational diabetes mellitus	p3134	increases	(98)
Gestational diabetes mellitus	ANRIL	increases	(98)
Gestational diabetes mellitus	AC092159.2	increases	(98)
Recurrent spontaneous abortion	NEAT1	decreases	(122)
Recurrent spontaneous abortion	MALAT	decreases	(114)
Recurrent spontaneous abortion	SNHG7	decreases	(120)
Recurrent spontaneous abortion	ANRIL	decreases	(122)
Recurrent spontaneous abortion	PVT1	decreases	(122)
Recurrent spontaneous abortion	HOTAIR	decreases	(121)
Recurrent spontaneous abortion	SNHG5	decreases	(119)
Recurrent spontaneous abortion	H19	increases	(113)
Recurrent spontaneous abortion	MEG8	increases	(122)
Recurrent spontaneous abortion	LINC01088	increases	(116)
Recurrent spontaneous abortion	SLC4A1-1	increases	(117)

## Conclusion

Exosomal lncRNAs have a wide range of potential for investigation (123). Exosomal lncRNAs have the power to control a wide range of cellular biological processes, including the recasting of the helical arteries, the inflammatory response, immunological control, cellular metabolism, and autophagy (124–126). Exosomal lncRNAs are more durable and less prone to degradation than serum-derived lncRNAs, allowing them to move unaltered from their “origin” to their “destination” and carry out their intended functions (127–130). Exosomal lncRNAs at the maternal-fetal interface have been shown in numerous studies to play an essential role in pregnancy-specific illnesses and to support embryo implantation and maintenance. Hence, it is necessary to address the issue of how to harvest exosomes that more accurately depict the cellular environment *in vivo* (126, 131, 132). Pregnancy-specific disorders have been linked to abnormal changes in placenta-derived exosomes seen in the peripheral blood of pregnant women, although larger sample sizes are still required to confirm their utility as biomarkers for clinical testing. Exosomes can also forecast embryonic developmental potential, and shortly, using exosomes as markers in clinical testing will be a promising and significant noninvasive test (133, 134).

## Author contributions

MW, LZ, SM, RL, JL, and SY performed literature searches and selected the studies and reviews discussed in the manuscript. The first draft of the manuscript was prepared by MW. LZ, SM, RL, and JL made subsequent amendments. SY revised the manuscript. All authors read and approved the final manuscript and contributed to the conception of this review.

## Glossary

**Table:**

ESE	early-sorting endosome
MVBs	multivesicular bodies
ESCRT	endosomal sorting complex required for transport
PLAP	placental-like alkaline phosphatase
PRC2	Polycomb repressive complex 2
HOTAIR	HOX antisense intergenic RNA
hnRNPA2B1	heterogeneous ribonucleoprotein A2B1
HLEC	human lymphatic vessel endothelial cells
DMR	differently methylated regions
SR	serine/arginine-rich
MALAT1	metastasis-associated lung adenocarcinoma transcript 1
PCR	polymerase chain reaction
RIF	recurrent implantation failure
ZEB2-AS1	Zinc finger E-box-binding homeobox 2 antisense RNA 1
PEXO	Placenta-derived exosome
HK2	recombinant hexokinase 2
HLAG	human leukocyte antigen G
MMP	matrix metalloproteinase
PS	phosphatidylserine
HUCMSCs	Human umbilical cord mesenchymal stem cells
VEGF	vascular endothelial growth factor
SNHG22	short nucleolar RNA host gene 22
EZH2	enhancer of zeste homolog 2
RND3	Rho family GTPase 3
DC	dendritic cell
NOMO1	nodal modulator 1
T2DM	diabetes mellitus type 2
IR	insulin resistance
NGT	normal glucose tolerance
PAPPA	pregnancy-associated plasma protein A
FBG	fasting blood glucose
HbA1c	glycosylated hemoglobin, type A1c
HOMA	homeostasis model assessment
MEG3	maternally expressed gene 3
HUVEC	human umbilical vein endothelial cells
SNX17	sorting nexin 17
HOTTIP	HOXA transcript expression at the distal tip
CDKs	cyclin-dependent kinases
URSA	unexplained recurrent spontaneous abortion
SIRT	silent information regulator
NEAT1	nuclear enriched transcript 1
PVT1	plasmacytomvariant translocation 1
